# Potential Therapeutic Mechanism of Scutellaria baicalensis Georgi against Ankylosing Spondylitis Based on a Comprehensive Pharmacological Model

**DOI:** 10.1155/2022/9887012

**Published:** 2022-12-21

**Authors:** Xu Li, Jian Liu, Yanyan Fang, Dan Huang, Mingyu He, Fanfan Wang, Qi Han

**Affiliations:** ^1^First School of Clinical Medicine, Anhui University of Chinese Medicine, Hefei, China; ^2^Department of Rheumatism Immunity, The First Affiliated Hospital, Anhui University of Chinese Medicine, Hefei, China; ^3^Department of Clinical Data Center, The First Affiliated Hospital, Anhui University of Chinese Medicine, Hefei, China

## Abstract

**Background:**

Scutellaria baicalensis Georgi (SBG) has significant anti-inflammatory and immune-modulating activities and is widely used in the treatment of inflammatory and autoimmune diseases. However, the mechanism of SBG in the treatment of ankylosing spondylitis (AS) remains to be elucidated.

**Methods:**

Differentially expressed genes (DEGs) related to AS were analyzed based on two GEO gene chips. The DEGs were merged with the data derived from OMIM, GeneCards, and PharmGKB databases to ascertain AS-related targets. Active components of SBG and their targets were acquired from the TCMSP database. After overlapping the targets of AS and SBG, the action targets were acquired. Subsequently, protein-protein interaction (PPI) network and core target screening were conducted using the STRING database and Cytoscape software. Moreover, the DAVID platform was used to perform Gene Ontology (GO) and Kyoto Encyclopedia of Genes and Genomes (KEGG) analyses of action targets. Finally, the affinity of major active components and core targets was validated with molecular docking.

**Results:**

A total of 36 active components of SBG were acquired from TCMSP database. Among these, the main active components were baicalein, wogonin, and oroxylin A. The PPI network and screening showed TNF, IL-6, CXCL8, PTGS2, and VEGFA as core targets associated SBG against AS. GO and KEGG analyses indicated that SBG participated in various biological processes, via regulating IL-17, TNF, and NF-*κ*B signaling pathways. Molecular docking results confirmed a strong binding activity between the main active components and the core targets.

**Conclusion:**

The therapeutic mechanism of SBG associated with AS can be characterized as a multicomponent, multitarget, and multipathway mechanism. SBG may be a promising therapeutic candidate for AS.

## 1. Introduction

Ankylosing spondylitis (AS), an autoimmune disorder, primarily afflicts the axial joint. Pathological changes mainly include inflammation, bone destruction, and new bone formation [1, 2]. This disease is significantly related to human histocompatibility leukocyte antigen B27 [3] along with causative factors such as infection, environment, genetics, and immunity [4]. AS has a global prevalence rate ranging from approximately 0.1% to 0.5% and primarily affects men, especially young men, with a male-to-female ratio of about 3 : 1 [5, 6]. In western medicine, AS is primarily treated via nonsteroidal anti-inflammatory drugs (NSAIDs), biological agents, and disease-modifying antirheumatic drugs. Nevertheless, the long-term use of these drugs may lead to adverse effects, resulting in poor patient compliance. Owing to its extensive history, considerable clinical experience has been accumulated with traditional Chinese medicine (TCM) in AS treatment.

TCM mainly treats AS by mediating the regulation of the immune inflammatory response. Scutellaria baicalensis Georgi (SBG) is the dry root of the Lamiaceae plant Huang-Qin [7]. Modern pharmacological studies have demonstrated an association of multiple pharmacological activities, including anti-inflammatory, antibacterial, and immune regulation, with its components [8–10]. Therefore, SBG is widely used to treat autoimmune and inflammatory diseases. However, TCM exerts its pharmacological effect via a comprehensive action of various components. Currently, the pharmacological effects and underlying mechanisms of SBG are primarily examined based on its single component; systematic research examining the biological basis of SBG in AS treatment is lacking. Network pharmacology can be utilized to clarify the overall effect of various compounds by comprehensively analyzing the composition of TCM, exploring the targets at the intersection of drugs and diseases, and analyzing target-related biological functions and pathways [11]. Such studies can help to lay a theoretical foundation for clinical trials [12]. Therefore, the study uses network pharmacology to explore the biological principle of SBG in AS treatment; its significance is not only to explore its key components and speculate its possible mechanism, to further understand the drug-target interaction mechanism provides important information, but also to provide a beneficial therapeutic strategy for the precise treatment of AS while providing a scientific basis for the development and clinical application of new drugs.

In this context, network pharmacology was utilized in our study to predict the active components, action targets, and pathways of SBG in AS treatment, thereby ascertaining the related mechanism. These results were preliminarily validated using molecular docking technology.

## 2. Materials and Methods

### 2.1. Study Design and Workflow

**Figure 1:**
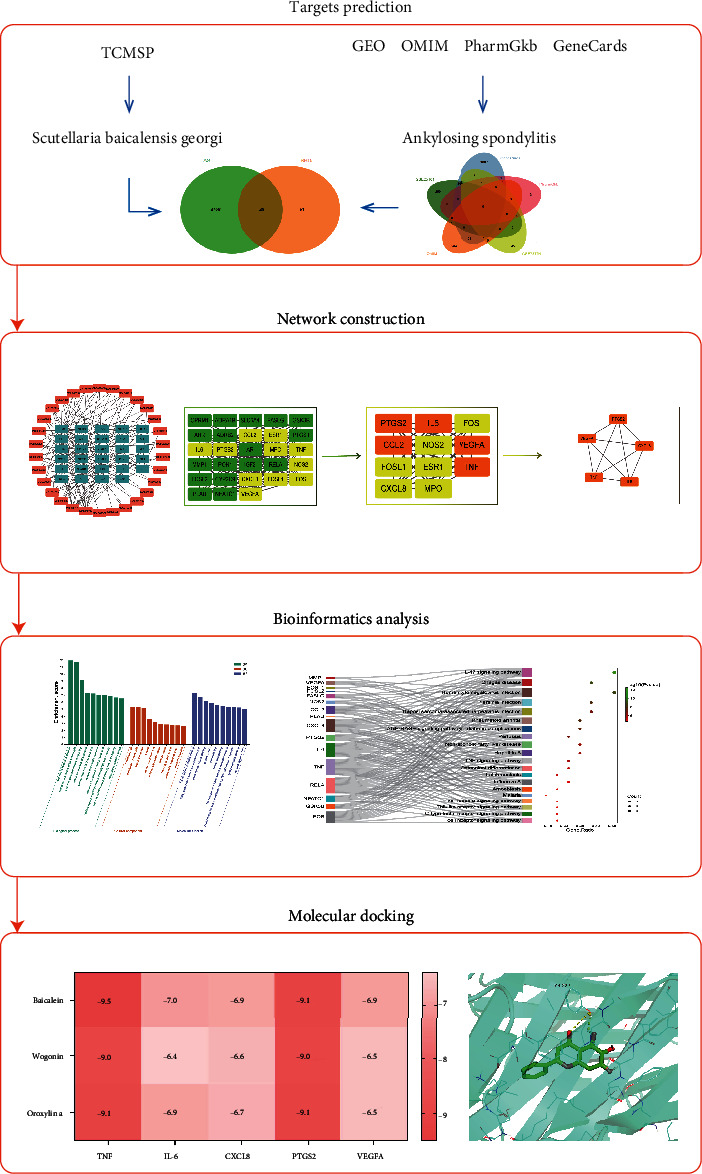
The workflow of the study.

The workflow of the current research is presented in Figure 1. Active components and targets of SBG and other AS-related targets were analyzed through database search, and a Venn diagram was used to integrate them to ascertain the targets of SBG in the treatment of AS. Then, the network of active component-action target and the protein-protein interaction (PPI) network of the action targets were constructed for further analysis. Gene Ontology (GO) and Kyoto Encyclopedia of Genes and Genomes (KEGG) analyses were performed to explore the biological functions and signaling pathways. Finally, the prediction results of the network pharmacology were verified by molecular docking.

### 2.2. Gene Microarray Screening

AS-related gene expression profile data were searched in the GEO database with the keyword “ankylosing spondylitis.” Data were included with the following criteria: datasets that included AS and normal samples and datasets with samples derived from *Homo sapiens* and the gene microarray data depicting gene expression profiles. Finally, two datasets (GSE25101 (including 16 AS samples and 16 normal samples) and GSE73754 (including 52 AS samples and 20 normal samples)) were selected for further analyses, and their corresponding matrices and platform annotation files were downloaded.

### 2.3. Microarray Data Analysis

R (https://www.r-project.org) was applied for systematic analyses. After the probes of raw data in AS-related microarray data were annotated and filtered, these raw data were subjected to probe averaging and background correction, and the samples were normalized using the normalize function. Subsequently, the combined data set was subjected to further analyses with the limma package, and the differentially expressed genes (DEGs) were retrieved under the conditions of *P* < 0.05 and |logFC| ≥ 1.3. The volcano plots and heat maps were drawn.

### 2.4. AS Target Pool

Using “ankylosing spondylitis” as the keyword, AS-related targets were searched in OMIM (https://omim.org/), GeneCards (https://www.genecards.org/), and PharmGKB (https://www.pharmgkb.org/) databases, which were intersected with the DEGs screened by gene microarray analyses to acquire the targets of AS. Thereafter, a Venn diagram of AS targets was drawn using the online platform jvenn [13].

### 2.5. Retrieval of the Active Components and Targets of SBG

The active components of SBG were searched in Traditional Chinese Medicine System Pharmacology (TCMSP) database [14]. Oral bioavailability (OB), a pivotal pharmacokinetic parameter of an oral drug, indicates the ratio between the oral dose and the systemic circulation of a drug [15]. Drug-likeness (DL), which refers to physical and chemical properties, is a qualitative principle for assessing whether a compound is similar to a known drug. The active components of SBG were screened with the following criteria: OB ≥ 30% and DL ≥ 0.18. Then, the targets of SBG were also obtained from the TCMSP. The resulting targets were input into the UniProt (https://www.uniprot.org/) for standardization of their names. jvenn was employed to match the AS targets with the targets of SBG to acquire the potential targets of SBG in AS treatment.

### 2.6. Construction of an Active Component-Action Target Network

The active components corresponding to the aforementioned action targets were mapped with Perl (https://www.perl.org/). Next, a network of “active component-action target” was constructed by importing the above components and targets of SBG into Cytoscape (version 3.9.1). Here, a node referred to the active components and action targets, and an edge marked the interaction of the active components with the action targets.

### 2.7. PPI Network Construction and Core Target Screening

The STRING database (https://STRING-db.org/) is used for searching protein interactions and for predicting protein interactions from many organisms [16]. Interactions among the action targets were analyzed using this database. The organism was set as *Homo sapiens*, and the minimum interaction score was set as 0.4. The isolated targets were hidden. A tsv format file of the protein interaction was acquired and exported. This file was then imported into Cytoscape for visualizing the PPI network, which was then topologically analyzed with the CytoNCA plug-in. The screening criteria were as follows: degree centrality and betweenness centrality higher than or equal to its median value. The screening was performed twice to obtain the core targets of SBG for AS treatment.

### 2.8. Bioinformatics Analysis

The biological function and specific pathways of potential targets were analyzed with the DAVID database (https://david.ncifcrf.gov/), which is widely utilized in gene annotation. Specifically, the action targets were inputted into the DAVID for GO and KEGG analyses. GO functional annotation was applied to annotate mechanisms associated with the targets with respect to biological process (BP), cellular components (CC), and molecular function (MF) [17] and analyze the top 10 entries in each section. Additionally, KEGG enrichment analysis was used to annotate the target-related pathways [18] and select the top 20 items to harvest the primary pathways of SBG involved in AS treatment. Then, R was employed to mark the targets associated with the most critical pathways that were enriched in KEGG analysis.

### 2.9. Molecular Docking

To verify whether the active components and action targets exhibit better binding activity, the top three major active components acquired from the active component-action target network were considered ligands, and the core targets obtained from the PPI network screening were considered receptors for molecular docking. The “mol2” format files of the major active components were downloaded from the TCMSP database. Likewise, the structure of the core targets was obtained from the PDB database (https://www.rcsb.org/). Thereafter, AutoDock (version 4.2.6, https://autodock.scripps.edu/) was used for molecular docking, and the results were drawn with PyMOL (version 2.5, https://pymol.org/2/). The binding affinity was calculated, thereby verifying the accuracy of the network pharmacological results. An affinity of <0 kcal/mol indicated that the ligand and the receptor could bind spontaneously, and a lower affinity represented a stronger binding force between the ligand and the receptor [19]. The absolute value of affinity of ≥5 kcal/mol indicated a preferable binding activity between the two, and the absolute value of affinity of ≥9 kcal/mol reflected an extremely strong binding activity.

## 3. Results

### 3.1. DEGs of AS

**Figure 2:**
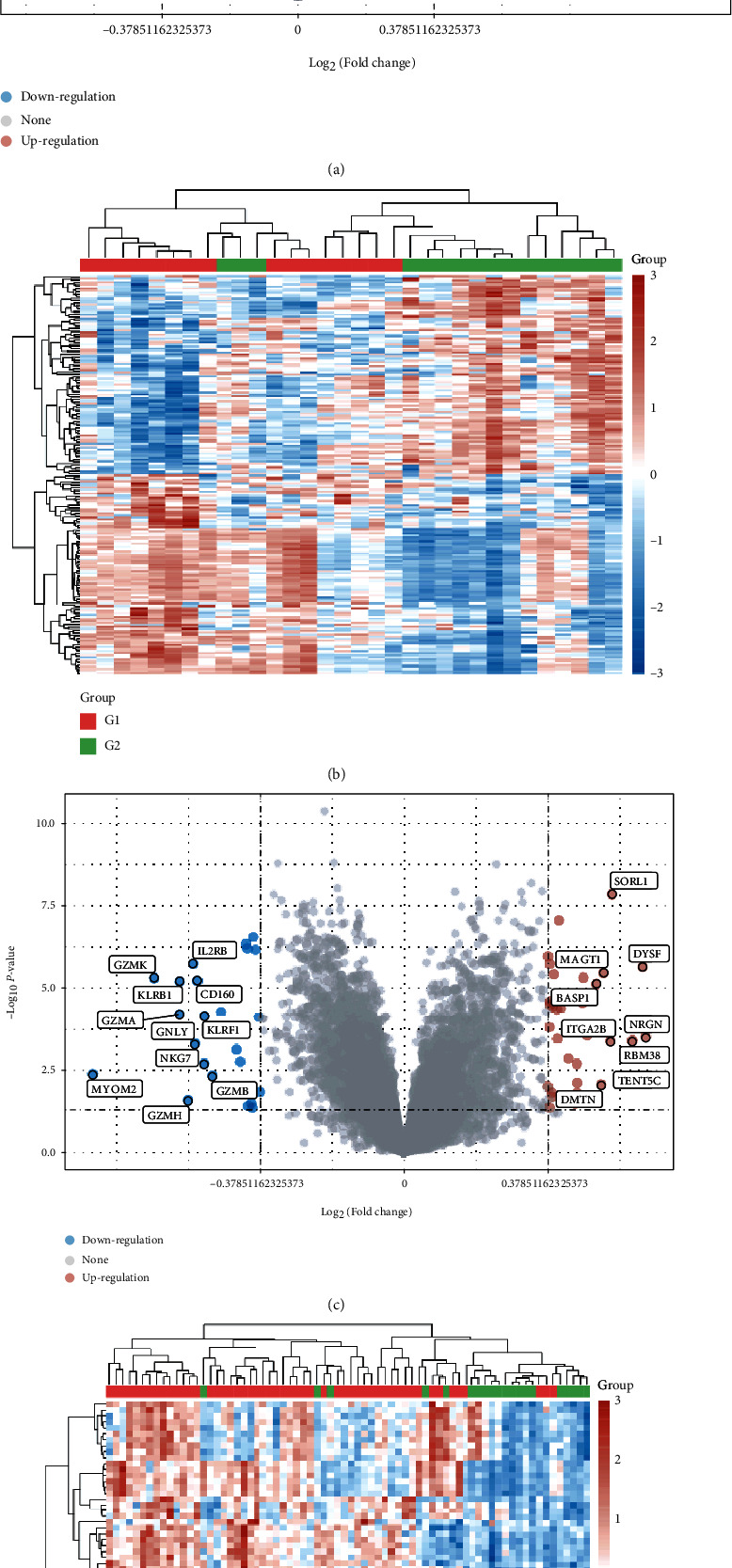
Volcano plots and heat maps of DEGs. (a) Volcano plots of GSE25101. (b) Heat maps of GSE25101. (c) Volcano plots of GSE73754. (d) Heat maps of GSE73754. In the volcano plots, red indicates upregulated genes, blue refers to downregulated genes, and gray stands for normal genes. In the heat maps, upregulated genes are displayed in red and downregulated genes in blue. AS samples are referred to as G1 and normal samples as G2.

Gene microarray data were sorted and analyzed using R. A total of 369 DEGs including 189 upregulated genes and 120 downregulated genes in GSE25101 and 37 upregulated genes and 23 downregulated genes in GSE73754 were derived. The volcano plots and heat maps were plotted using R (Figure 2).

### 3.2. AS Target Pool

**Figure 3:**
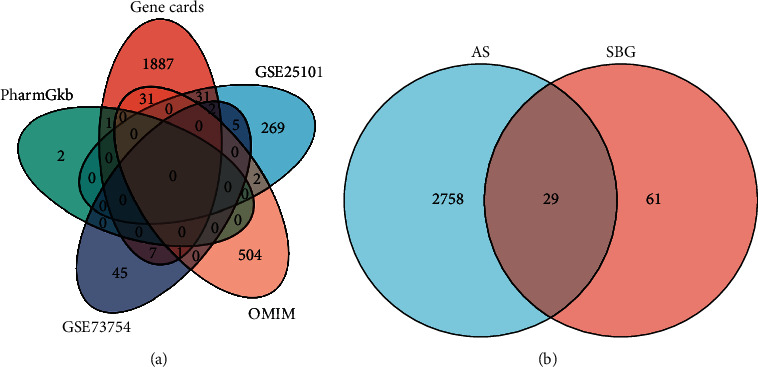
Venn diagram. (a) The Venn diagram of AS-related targets. (b) The Venn diagram of the overlapping targets of SBG and AS.

A total of 1960 targets were retrieved from GeneCards, 538 targets from OMIM, and 3 targets from PharmGKB. Then, 2787 targets of AS were derived upon the combination with DEGs and the deletion of duplicate targets (Figure 3(a)).

### 3.3. Active Components and Targets of SBG

In TCMSP, 36 active components of SBG were acquired (Table 1), corresponding to 90 targets. The intersection of these targets with AS targets yielded 29 action targets of SBG that were involved in the AS treatment (Figure 3(b)).

### 3.4. Network of Active Component-Action Targets

**Figure 4:**
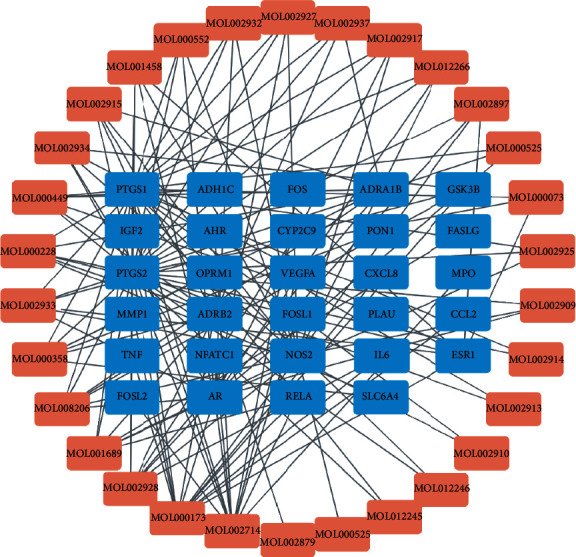
Active component-action target network. Light red nodes signify the active components of SBG, and blue nodes refer to the targets of SBG involved in the treatment of AS. The lines represent the active component acting on the corresponding targets, with the top three targets being MOL002714 (baicalein), MOL000173 (wogonin), and MOL002928 (oroxylin A) with the most lines.

A network of active component-action target was generated with Cytoscape (Figure 4), including 29 action targets and the corresponding active components. In the network, the same active component acted on different targets, and the same target corresponded to diverse components, suggesting that SBG had the characteristics of multitarget and multicomponent synergy for AS treatment. Lines denoted corresponding relationships of the active components with the action targets. As depicted in Figure 4, the top three targets MOL002714, MOL000173, and MOL002928 showed the most lines and corresponded with the following active components: baicalein, wogonin, and oroxylin A. These were considered as the major active components of SBG involved in AS treatment.

### 3.5. PPI Network and Screening of Core Targets

**Figure 5:**
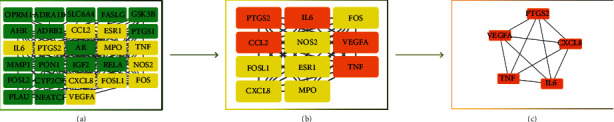
Identification of core targets. (a) PPI network of the action targets. (b) PPI network derived after the first screening. (c) PPI network derived after the second screening. Nodes stand for action targets, and edges illustrate interactions between targets and targets. After screening twice, TNF, IL-6, CXCL8, PTGS2, and VEGFA were ascertained as the core targets.

We imported 29 targets into the STRING to acquire the PPI network, and the network was visualized by Cytoscape. The network had 28 nodes and 153 edges after removing the free ones (Figure 5(a)). Next, screening was conducted twice with the CytoNCA plug-in to obtain the interaction network of core targets, which included TNF, IL-6, CXCL8, PTGS2, and VEGFA (Figure 5(c)). These five core targets were identified to play a vital role in AS treatment with SBG and, hence, were selected for subsequent verification.

### 3.6. Bioinformatics Analysis

**Figure 6:**
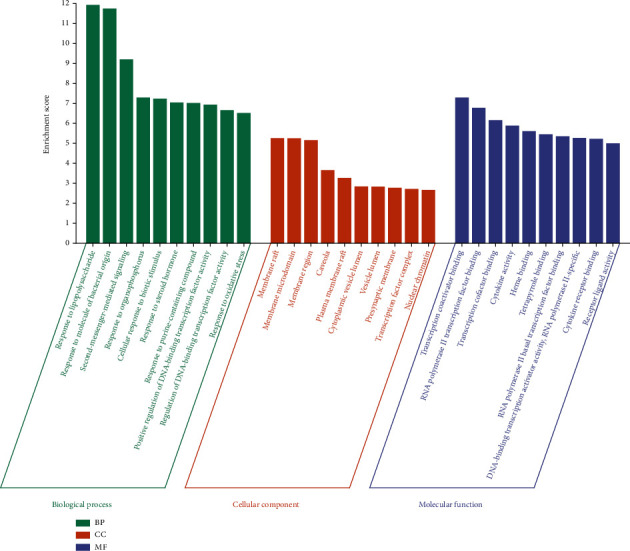
Bar plot of GO analysis. The top 10 GO entries for biological processes, cellular components, and molecular functions were selected (*P* < 0.05). The horizontal axis represents the GO entries, and the vertical axis indicates the number of genes enriched with each item.

**Figure 7:**
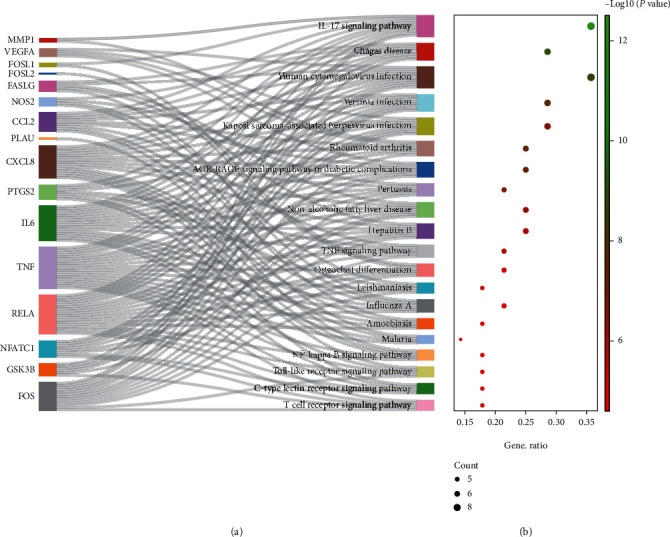
The Sankey and Bubble plot of KEGG analysis. (a) The Sankey plot represents the enriched targets in each pathway, and (b) indicates the bubble plot. The greener the bubble, the higher the enrichment value.

**Figure 8:**
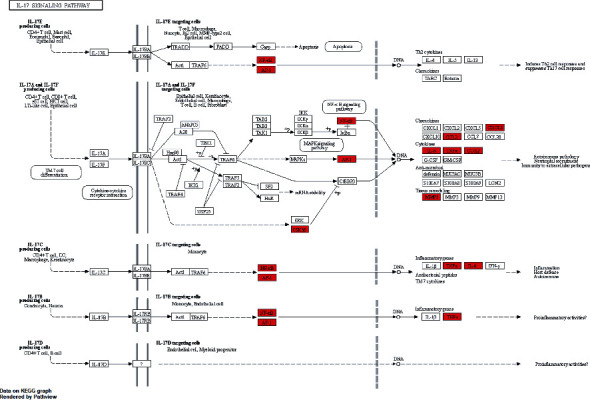
IL-17 pathway map. The relevant targets of SBG in the IL-17 pathway involved in AS treatment are exhibited in red nodes.

A total of 29 action targets were imported into DAVID for bioinformatics analysis. Specifically, these targets were considerably enriched in 1385 GO entries, including 1285 BPs (majorly including responses to lipopolysaccharides, responses to molecules of bacterial origin, and second-messenger-mediated signaling), 26 CCs (mainly involving membrane raft, membrane microdomain, and membrane region), and 74 MFs (primarily protein binding, enzyme binding, and receptor binding). The top 10 targets of each entry are demonstrated in Figure 6. Additionally, these targets were enriched in 93 pathways. These pathways primarily involved the IL-17 pathway, TNF pathway, and NF-kappa B pathway, which were closely related to AS. The top 20 pathways are exhibited in Figure 7. The most crucial IL-17 pathway map was derived from the KEGG database. The specific action sites of targets in this pathway have been indicated in red in Figure 8.

### 3.7. Molecular Docking

**Figure 9:**
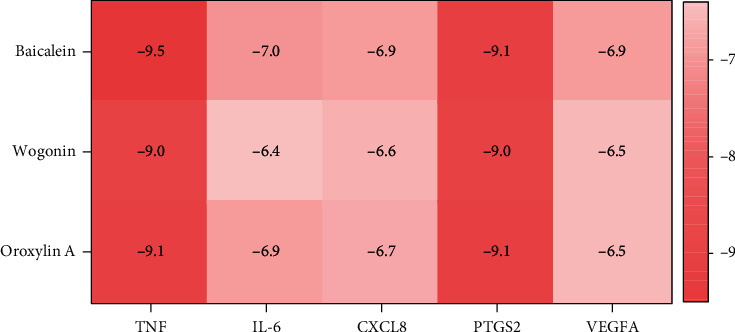
Heat map of docking scores for three major active components with five core targets. The redder the color, the stronger the binding activity.

**Figure 10:**
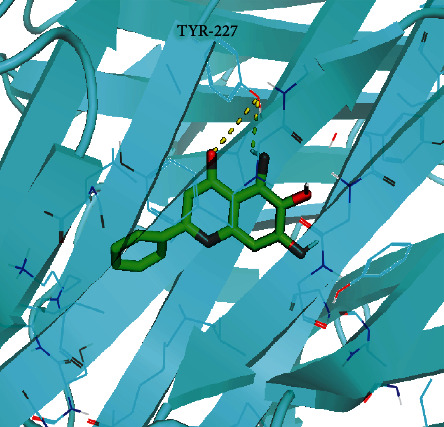
Molecular docking of baicalein with TNF.

We obtained three major active components (baicalein, wogonin, and oroxylin A) from the active component-action target network and five core targets (TNF, IL-6, CXCL8, PTGS2, and VEGFA) from PPI network screening for molecular docking. And the docking affinity is presented in Figure 9. The affinity values were all <-5 kcal/mol; baicalein and TNF showed the best binding activity (Figure 10). The results indicated that the major active components of SBG showed good binding to the core targets, which supported the reliability of the network pharmacology results.

## 4. Discussion

AS is an autoimmune disease characterized by inflammation, and its pathogenesis has not yet been fully clarified [20]. At present, no specific drug is available for this disease; hence, it is necessary to find effective and safe anti-AS drugs. Network pharmacology integrates multiple disciplines, such as system biology and data analysis [21, 22], emphasizing the multichannel regulation of signaling pathways. It is suitable for elucidating the mechanisms of action of multiple chemical components and molecular targets [23], and hence, it is a credible approach for modern research in TCM. The GEO database provides a critical online resource for researchers to deeply mine gene expression data [24, 25]. Compared with ordinary gene expression profiling studies, multimicroarray conjoint analysis integrates the raw data of multiple microarrays to augment the accuracy and reliability of results. Multidisciplinary cross-integration has led to a new stage in pharmacology research in TCM with rapid development. Hence, we aimed to examine the active components of SBG in AS treatment and the potential mechanism of action by combining the aforementioned technologies according to the research idea of multicomponent and multitarget synergy.

Inflammation, bone destruction, and new bone formation are the main pathological characteristics of AS, which are closely related to abnormal immune inflammatory reactions. Therefore, inhibiting immune inflammatory reaction is an important strategy to control the progression of AS. Interestingly, in this study, baicalein, wogonin, and oroxylin A were discovered as the major active components of SBG in AS treatment after searching for all the active components and analyzing the active component-action target network. The aforementioned main active components had anti-inflammatory and immunomodulatory activities. Baicalein and wogonin are the principal flavonoid active compounds present in SBG [26]. Baicalein demonstrates an extensive range of pharmacological effects, such as antioxidant, immunomodulatory, antiviral, free radical scavenging, anti-inflammatory, and antipyretic effects [27–29]. Baicalein orchestrates the arachidonic acid metabolic pathway and suppresses the production of cyclooxygenase, deoxygenase, nuclear factors, and cytokines, thereby exhibiting a potent antipyretic and analgesic effect [30]. Wogonin performs various biological functions, including anti-inflammatory, antiallergic, antioxidant, and antiapoptotic functions [31, 32], and its anti-inflammatory activity may be associated with a reduction in iNOS and COX-2 expression [33]. Oroxylin A also has anti-inflammatory and antiallergic effects. It represses inflammation to curb the binding and transcriptional activation of the nuclear transcription factor NF-*κ*B, thereby reducing the expression of iNOS and COX-2 [34]. Therefore, SBG contains a variety of active components with anti-inflammatory activities, reflecting “multicomponent” characteristics.

The study revealed that TNF, IL-6, CXCL8, PTGS2, and VEGFA may be core targets of SBG involved in the treatment of AS. These targets are closely associated with immune inflammation. In particular, TNF-*α*, a classic proinflammatory factor, can mediate an inflammatory cascade and promote the upregulation of various inflammatory factors, such as IL-6, IL-1*β*, and IL-8 [35]. It also can directly stimulate inflammatory cells and catalyze inflammatory reactions to induce tissue cell damage [36, 37], which is closely related to the pathological changes in AS. Clinically, biologics targeting TNF-*α* are important drugs used in western medicine for the treatment of AS. These drugs primarily alleviate AS by inhibiting inflammation and slowing the progression of spinal imaging. As a pleiotropic cytokine with multiple biological functions, IL-6 participates in the pathogenesis of various rheumatic disorders [38]. It promotes AS fibroblast ossification via MAPK/ERK signaling [39]. CXCL8, also known as the IL-8, is synthesized by macrophages and epithelial cells in response to a variety of stimuli. Higher levels of CXCL8 have been observed in patients with AS [40]. It is also a typical inflammatory cytokine that recruits and activates immune cells, thereby leading to a local inflammatory response [41]. PTGS2, also known as COX-2, is an inducible enzyme that mediates prostaglandin (PG) synthesis, the upregulation of which leads to the proliferation of fibroblast-like synoviocytes and aggravation of inflammation, further promoting AS [42]. Interestingly, in the study, we found that COX-2 is the core target of SBG involved in the treatment of AS. As the first-line drugs used for treating AS, NSAIDs also reduce the production of PG mainly by inhibiting the activity of COX-2, thereby demonstrating an anti-inflammatory effect. This suggests that SBG and NSAIDs may have similar anti-inflammatory pathways. As a proangiogenic factor, VEGFA is involved in angiogenesis, and it is closely related to the pathogenesis of inflammatory diseases. Furthermore, the increase in serum VEGFA levels accelerates AS development by disrupting the balance of bone metabolism, repressing bone resorption, and enhancing bone formation [43]. Thus, the role of these core targets in the pathological process of AS is closely related to the immune inflammatory response. The results of molecular docking also showed that the affinity value of the main active components and the core targets was less than -5 kcal/mol, which strongly confirmed their good docking performance. Therefore, our study found that the treatment of AS by SBG involved a “multitarget” approach, which may ameliorate AS by modulating the immune-inflammatory response.

GO and KEGG analyses showed that the therapeutic effect of SBG may be related to responses to lipopolysaccharides, responses to molecules of bacterial origin, and IL-17, TNF, and NF-*κ*B signaling pathways. Lipopolysaccharide, which is a component of the bacterial cell wall, can stimulate the immune inflammatory response of the body and mediate the aggregation of adhesion molecules, cell chemokines, and inflammatory cytokines in local joints. It has been used to induce AS in animal models [44]. A previous study examining the etiology of AS suggests that intestinal bacteria may play an important role in AS pathogenesis [45]. Hence, responses to the molecule of bacterial origin may be a method for SBG to treat AS. Several clinical studies have demonstrated that TNF and IL-17 signaling are central to the pathogenesis of AS [46] and that NF-*κ*B pathway is involved in the formation of a hypercoagulable state in AS patients [47]. The proinflammatory cytokine, IL-17, can enhance the production of other proinflammatory cytokines, including TNF-*α* [48]. NF-*κ*B promotes inflammation and immune response and participates in apoptosis [49], playing an important role in AS disease. SBG may treat AS through multiple biological process and pathways, which reflects its “multipathway” characteristics.

Based on network pharmacology, this study analyzed the molecular mechanism of SBG in AS treatment, demonstrated the complex molecular network associated with SBG in AS treatment, and the characteristics of “multicomponent, multitarget, and multipathway” of SBG, providing a scientific basis for in-depth research and development of SBG as a drug for the treatment of AS. However, one of the main limitations of the study is that our findings are based on existing databases. Therefore, our results need to be further verified in cell experiments and clinical trials. In future research, we aim to group different active components of SBG, screen the active components with good therapeutic effects, and actively explore the optimal dosage of SBG for the treatment of AS along with possible adverse reactions. We aim to find a single component or a combination for the optimum treatment of AS and clarify the mechanism of action so that it can be applied to clinical practice.

## 5. Conclusion

The study suggests that the therapeutic mechanism of SBG associated with AS can be characterized as a multicomponent, multitarget, and multipathway mechanism. Baicalein, wogonin, and oroxylin A may be the major active components of SBG in AS treatment. These components primarily act on the core targets of TNF, IL-6, CXCL8, PTGS2, and VEGFA and regulate the IL-17, TNF, and NF-*κ*B signaling pathways, assuming a vital role in AS treatment. As a medicinal plant, SBG is hopeful to be developed as an effective drug for the treatment of AS.

## Figures and Tables

**Table 1:** Active components of SBG.

Mol ID	Molecule name	OB	DL
MOL001689	Acacetin	34.97	0.24
MOL000173	Wogonin	30.68	0.23
MOL000228	(2R)-7-Hydroxy-5-methoxy-2-phenylchroman-4-one	55.23	0.2
MOL002714	Baicalein	33.52	0.21
MOL002909	5,7,2,5-Tetrahydroxy-8,6-dimethoxyflavone	33.82	0.45
MOL002910	Carthamidin	41.15	0.24
MOL002913	Dihydrobaicalin_qt	40.04	0.21
MOL002914	Eriodyctiol (flavanone)	41.35	0.24
MOL002915	Salvigenin	49.07	0.33
MOL002917	5,2′,6′-Trihydroxy-7,8-dimethoxyflavone	45.05	0.33
MOL002925	5,7,2′,6′-Tetrahydroxyflavone	37.01	0.24
MOL002927	Skullcapflavone II	69.51	0.44
MOL002928	Oroxylin a	41.37	0.23
MOL002932	Panicolin	76.26	0.29
MOL002933	5,7,4′-Trihydroxy-8-methoxyflavone	36.56	0.27
MOL002934	Neobaicalein	104.34	0.44
MOL002937	Dihydrooroxylin	66.06	0.23
MOL000358	Beta-sitosterol	36.91	0.75
MOL000359	Sitosterol	36.91	0.75
MOL000525	Norwogonin	39.4	0.21
MOL000552	5,2′-Dihydroxy-6,7,8-trimethoxyflavone	31.71	0.35
MOL000073	ent-Epicatechin	48.96	0.24
MOL000449	Stigmasterol	43.83	0.76
MOL001458	Coptisine	30.67	0.86
MOL001490	Bis[(2S)-2-ethylhexyl] benzene-1,2-dicarboxylate	43.59	0.35
MOL002879	Diop	43.59	0.39
MOL002897	Epiberberine	43.09	0.78
MOL008206	Moslosooflavone	44.09	0.25
MOL010415	11,13-Eicosadienoic acid, methyl ester	39.28	0.23
MOL012245	5,7,4′-Trihydroxy-6-methoxyflavanone	36.63	0.27
MOL012246	5,7,4′-Trihydroxy-8-methoxyflavanone	74.24	0.26
MOL012266	Rivularin	37.94	0.37
MOL002908	5,8,2′-Trihydroxy-7-methoxyflavone	37.01	0.27
MOL002911	2,6,2′,4′-Tetrahydroxy-6′-methoxychaleone	69.04	0.22
MOL002926	Dihydrooroxylin A	38.72	0.23
MOL001506	Supraene	33.55	0.42

## Data Availability

All the datasets will be available upon contacting the corresponding author.
